# Alanine Aminotransferase within Reference Range Is Associated with Metabolic Syndrome in Middle-Aged and Elderly Chinese Men and Women

**DOI:** 10.3390/ijerph111212767

**Published:** 2014-12-10

**Authors:** Xuebing Zhang, Yiming Mu, Wenhua Yan, Jianming Ba, Hongmei Li

**Affiliations:** 1 Department of Endocrinology, Chinese People’s Liberation Army (PLA) General Hospital, Beijing 100853, China; E-Mails: snowicechina@hotmail.com (X.Z.); yanwenhua301@163.com (W.Y.); jmba86@yahoo.com (J.B.); 2 Department of Endocrinology, MeiTan General Hospital, Beijing 100028, China; E-Mail: lihongmei64@163.com

**Keywords:** metabolic syndrome, alanine aminotransferase, middle-aged and elderly

## Abstract

*Objective*: To investigate the association between serum ALT level within reference range (≤40 U/L) and morbidity of MetS in a large middle-aged and elderly Chinese community population. *Methods*: Our study was a community-based cross-sectional survey which used cluster sampling method. From November 2011 to August 2012 a total of 16,539 subjects (males 5184; females 11,355) with serum ALT levels in the normal range aged ≥40 years from Shijingshan District (Beijing, China) were included in the study. Data on demographic information, lifestyle, history of diabetes mellitus, hypertension, dyslipidemia and liver disease were collected. Body height, body weight, waist circumference, hip circumference, and blood pressure were recorded. The oral glucose tolerance test or a standard meal test and blood lipid test was performed. The determination of metabolic syndrome was according to the unified criteria published in 2009. The association between serum ALT level and metabolic syndrome was evaluated by logistic regression. The association between serum ALT level and all components of metabolic syndrome was evaluated by multiple linear regression. *p* < 0.05 was regarded as statistically significant. *Results*: The prevalence of metabolic syndrome was 41.4% in males and 40.6% in females. We found ALT level was positively associated with odds of metabolic syndrome after adjustment for age, smoking, and alcohol intake. The odds ratio values of MetS in the ALT quartiles 2–4 groups were 1.920 (95%CI: 1.619–2.277), 2.853 (95%CI: 2.407–3.381), and 4.171 (95%CI: 3.510–4.956) in males; 1.889 (95%CI: 1.671–2.136), 3.490 (95%CI: 3.095–3.935), and 5.593 (95%CI: 4.957–6.311) in females, respectively, compared with the ALT quartile 1 group. *Conclusions*: Higher serum ALT level within the reference range was associated with increased odds of MetS in middle-aged and elderly Chinese men and women.

## 1. Introduction

Metabolic syndrome (MetS) is a group of disorders, including obesity, hyperglycemia, dyslipidemia, and hypertension. Metabolic syndrome is strongly associated with development of type 2 diabetes mellitus and cardiovascular disease [1,2,3,4,5].

Non-alcoholic fatty liver disease (NAFLD), which is characterised by a wide spectrum of liver pathologies ranging from simple liver steatosis to non-alcoholic steatohepatitis to liver fibrosis to cirrhosis [6], is associated with characteristics of MetS such as insulin resistance (IR) and obesity and is considered as the hepatic component of MetS [7,8]. The liver enzyme alanine aminotransferase (ALT) is most closely related to liver fat accumulation [9], and is commonly used as marker of NAFLD [10]. Several cross-sectional studies have demonstrated that elevated ALT level is associated with MetS [11,12,13,14,15,16,17,18]. A recent meta-analysis based on prospective epidemiological data also shown that ALT was strongly correlated with incident MetS [19]. However, in the majority of uncomplicated obesity and NAFLD cases, ALT is still in the normal range [20,21]. At present, there are few studies on the association between ALT within the normal range and MetS in China. Therefore, the present study aimed to investigate the association between serum ALT level within reference range and morbidity of MetS in a large-scale middle-aged and elderly Chinese community population.

## 2. Methods

### 2.1. Study Population

The study population came from an ongoing longitudinal study (REACTION) that was designed to investigate the relationship between type 2 diabetes, pre-diabetes and the risk of cancer in the Chinese population [22]. This present study was a community-based cross-sectional survey which used cluster sampling method. We conducted this survey in Shijingshan District, Beijing, China from November 2011 to August 2012. A total of 21,428 permanent residents aged 40 and older in three urban communities were invited by telephone or door-to-door visit to participate in the study. The subjects which refused to participate this survey, had poor health, and were difficult to communicate with were excluded (*n* = 2154). From them, 19,274 residents (male 6784; female 12,490) signed the informed consent before the investigation. The response rate was 89.9%. The subjects who had incomplete demographic information, no availability of fasting blood glucose or 2-hour blood glucose (*n* = 129) were excluded, as were those with excessive alcohol intake (>80 g/d; *n* = 36), those with a history of virus hepatitis, liver carcinoma, liver cirrhosis, autoimmune liver disease, hyperthyroidism or schistosomiasis (*n* = 211). In addition, we also excluded the individuals with serum ALT level greater than 40 U/L (*n* = 2359). Finally, 16,539 individuals with ALT in the reference range (≤40 U/L) were included in our study. Our study was supported by Chinese PLA General Hospital Ethics Committee.

### 2.2. Clinical Data and Biochemical Indicators

We used a standard questionnaire to collect clinical data including demography, lifestyle, previous history of diabetes, hypertension, dyslipidemia and liver disease by trained physicians. The occupation (worker, soldier, cadre, technician, medical worker, teacher, individual trader, service provider, materfamilias, retiree, unemployed people) and education degree (illiterate, semi-literate, elementary, junior, senior, college) were also collected. Procedure of physical examination, tests of biochemical indicators, and performance of 75 g OGTT or standard meal test were already described in detail in a previous publication [23].

### 2.3. Definition

The determination of metabolic syndrome was according to the unified criteria published in 2009 [24]. Abdominal obesity was defined as waist circumference of ≥85 cm in males or ≥80 cm in females. TG concentration of ≥1.7 mmol/L, HDL-C concentration of <1.0 mmol/L in males or HDL-C concentration of <1.3 mmol/L in females were defined as dyslipidemia. Systolic blood pressure of 130 mmHg or higher, diastolic blood pressure of 85 mmHg or higher, or taking antihypertensive agents, were defined as hypertension. Fasting blood glucose level of 5.6 mmol/L or higher, or taking antidiabetic agents were defined as hyperglycemia. In China, upper normal limit of ALT is 40 U/L. Smoking was defined as smoking one or more cigarettes daily for at least a half year. Regular drinking was defined as at least once a week for a half year.

### 2.4. Statistical Analysis 

Statistical analysis was performed on SPSS software version 16.0 (SPSS Inc., CGO, IL, USA). Variables were presented as means ± SD, or percentage. The distribution of ALT was skewed, log transformation was used. Linear regression analysis was used to test for the trend across the four ALT quartile groups. Logistic regression was used to evaluate the association between serum ALT level and metabolic syndrome. The association between serum ALT level and all components of metabolic syndrome was evaluated by multiple linear regression. Log (ALT) was regarded as the dependent variable. Age, BMI, waist circumference, SBP, DBP, TC, TG, HDL-C, LDL-C, and fasting blood glucose as independent variables entered into the multiple linear regression model. *p* value of <0.05 was regarded as statistical significance.

## 3. Results 

The general characteristics of all men and women were presented in Table 1. The prevalence of metabolic syndrome was 41.4% in males and 40.6% in females.

**Table 1:** General characteristics of population.

Variables	Males (*n* = 5184)	Females (*n* = 11,355)
Age (years)	60.3 ± 8.5	57.2 ± 8.1
BMI (Kg/m^2^)	25.6 ± 3.2	25.5 ± 4.8
Waist circumference (cm)	86.8 ± 8.1	81.7 ± 8.8
SBP (mmHg)	134.5 ± 16.8	130.0 ± 16.7
DBP (mmHg)	77.4 ± 10.1	74.1 ± 9.8
TC (mmol/L)	4.91 ± 0.93	5.33 ± 0.99
TG (mmol/L)	1.56 ± 1.23	1.52 ± 0.98
HDL-C (mmol/L)	1.30 ± 0.34	1.49 ± 0.37
LDL-C (mmol/L)	3.02 ± 0.77	3.26 ± 0.83
FBG (mmol/L)	6.15 ± 1.92	5.85 ± 1.61
ALT (U/L)	23.5 ± 7.5	18.8 ± 7.1
Smoking (%)	41.5	2.1
Drinking (%)	22.3	1.2
Metabolic syndrome (%)	41.4	40.6

BMI, body mass index; SBP, systolic blood pressure; DBP, diastolic blood pressure; TC, total cholesterol; TG, triglyceride; HDL-C, high-density lipoprotein cholesterol; LDL-C, low-density lipoprotein cholesterol; FBG, fasting blood glucose; ALT, alanine aminotransferase.

We analyzed the association between serum ALT level and metabolic syndrome separately in males and in females. The study population was divided into four groups according to ALT quartile. The serum ALT quartile was as follows: <17.7 U/L, 17.7–22.6 U/L, 22.7–28.8 U/L, 28.9–40 U/L in males; <13.6 U/L, 13.6–17.2 U/L, 17.3–22.6 U/L, 22.7–40 U/L in females. Subjects in the highest ALT quartile group were more likely to be obese, had higher blood pressure, had higher TC, TG, LDL-C, fasting blood glucose level, and had lower HDL-C level than those of subjects in the lowest ALT quartile group in both men and women. In males, subjects in the highest ALT quartile group were likely younger than those in the lowest quartile; oppositely, in females, subjects in the lowest ALT quartile group were likely younger than those in the other three groups (Table 2 and Table 3).

**Table 2:** Characteristics of participants stratified by ALT quartiles in males.

Variables	Quartile 1	Quartile 2	Quartile 3	Quartile 4 ^*^
Metabolic syndrome (%)	25.4	38.2	46.7	55.0
ALT (U/L)	14.4 ± 2.4	20.0 ± 1.4	25.5 ± 1.8	33.8 ± 3.2
Age (years)	62.0 ± 8.8	60.6 ± 8.5	59.6 ± 8.3	58.8 ± 8.2
BMI (Kg/m^2^)	24.5 ± 3.2	25.4 ± 3.0	25.9 ± 3.0	26.5 ± 3.2
Waist circumference (cm)	84.1 ± 7.9	86.4 ± 7.9	87.8 ± 8.0	89.1 ± 7.8
SBP (mmHg)	132.7 ± 16.9	134.0 ± 16.9	135.0 ± 16.9	136.3 ± 16.5
DBP (mmHg)	75.4 ± 9.8	76.6 ± 10.1	78.2 ± 10.0	79.2 ± 9.9
TC (mmol/L)	4.70 ± 0.91	4.86 ± 0.87	4.93 ± 0.91	5.13 ± 0.97
TG (mmol/L)	1.19 ± 0.92	1.45 ± 1.08	1.64 ± 1.18	1.93 ± 1.53
HDL-C (mmol/L)	1.37 ± 0.34	1.31 ± 0.33	1.27 ± 0.33	1.25 ± 0.35
LDL-C (mmol/L)	2.86 ± 0.74	2.99 ± 0.73	3.05 ± 0.78	3.16 ± 0.80
FBG (mmol/L)	5.97 ± 1.75	6.07 ± 1.89	6.23 ± 2.05	6.32 ± 1.96

^*^ *p* for trend was significant for all variables (<0.05) by regression analysis.

**Table 3:** Characteristics of participants stratified by ALT quartiles in females.

Variables	Quartile 1	Quartile 2	Quartile 3	Quartile 4 ^#^
Metabolic syndrome (%)	20.6	33.9	48.5	59.2
ALT (U/L)	11.3 ± 1.8	15.4 ± 1.1	19.7 ± 1.6	28.8 ± 4.8
Age (years)	55.9 ± 8.4	57.4 ± 8.2	58.0 ± 7.9	57.7 ± 7.6
BMI (Kg/m^2^)	24.1 ± 3.3	25.2 ± 3.5	26.1 ± 3.1	26.7 ± 3.8
Waist circumference (cm)	78.3 ± 7.9	80.9 ± 8.5	83.0 ± 8.7	84.7 ± 8.7
SBP (mmHg)	126.0 ± 16.3	129.5 ± 16.4	131.8 ± 16.4	132.7 ± 16.9
DBP (mmHg)	72.2 ± 9.1	73.8 ± 9.3	74.8 ± 9.4	75.6 ± 11.0
TC (mmol/L)	5.11 ± 0.92	5.28 ± 0.96	5.41 ± 0.99	5.51 ± 1.04
TG (mmol/L)	1.16 ± 0.65	1.40 ± 0.79	1.64 ± 1.04	1.87 ± 1.18
HDL-C (mmol/L)	1.60 ± 0.37	1.52 ± 0.36	1.46 ± 0.35	1.40 ± 0.35
LDL-C (mmol/L)	3.06 ± 0.75	3.22 ± 0.79	3.35 ± 0.84	3.41 ± 0.87
FBG (mmol/L)	5.52 ± 1.27	5.70 ± 1.42	5.98 ± 1.72	6.18 ± 1.89

**^#^** *p* for trend was significant for all variables except age (<0.05) by regression analysis.

Higher ALT level was associated with higher prevalence of metabolic syndrome. After adjustment for age, smoking status, alcohol intake, occupation, and education degree, we found that odds of metabolic syndrome was higher in the subjects with the highest ALT quartile than those with the lowest ALT quartile (OR4.171, 95%CI: 3.510–4.956 for males; 5.593, 4.957–6.311 for females) (Table 4). In addition, after adjustment for age, BMI, TC, and LDL-C, ALT level was positively correlated with waist circumference, TG level, and fasting blood glucose in males; in females ALT level was positively associated with waist circumference, TG level, DBP, and fasting blood glucose (Table 5).

In addition, we drew the ROC curve according to serum ALT level and morbidity of MetS. Serum ALT level when the sum of sensitivity and specificity reached the maximum value was regarded as the optimal cutoff value. In males, the area under ROC curve was 0.638 ± 0.008.

**Table 4:** Multiple logistic regression for metabolic syndrome.

ALT Range	Males (*n* = 5184)			Females (*n* = 11,355)		
	OR	95%CI	*p*	OR	95%CI	*p*
Quartile 1	1	--	--	1	--	--
Quartile 2	1.920	1.619–2.277	<0.001	1.889	1.671–2.136	<0.001
Quartile 3	2.853	2.407–3.381	<0.001	3.490	3.095–3.935	<0.001
Quartile 4	4.171	3.510–4.956	<0.001	5.593	4.957–6.311	<0.001

**Table 5:** Multiple linear regression for all components of metabolic syndrome.

Variables	Males		Females	
	β	*p*	β	*p*
Waist circumference	0.103	<0.001	0.164	<0.001
TG	0.148	<0.001	0.204	<0.001
HDL-C	0.009	0.690	0.008	0.610
Systolic BP	0.033	0.073	0.019	0.102
Diastolic BP	0.024	0.201	0.042	<0.001
Fasting blood glucose	0.049	<0.001	0.084	<0.001

The optimal cutoff value of ALT level was 21.4 U/L. The sensitivity and specificity for diagnosis of MetS respectively was 68.2% and 52.7%. In females, the area under ROC curve was 0.679 ± 0.005. The optimal cutoff value of ALT level was 16.0 U/L. The sensitivity and specificity for diagnosis of MetS respectively was 74.1% and 52.5%.

## 4. Discussion 

Our results show that subjects in the highest ALT quartile group were more likely to be obese, have higher blood pressure, have higher TC, TG, LDL-C, fasting blood glucose level, and have lower HDL-C level. Subjects in the highest ALT quartile group were more prone to have metabolic syndrome. Furthermore, ALT level was positively correlated with waist circumference, TG level, and fasting blood glucose in males; in females ALT level was positively associated with waist circumference, TG level, DBP, and fasting blood glucose.

It has been demonstrated that NAFLD is associated with obesity and insulin resistance. Therefore, NAFLD is considered as the hepatic manifestation of MetS [7,8]. NAFLD is the most common cause of elevated ALT level in the developed countries [25,26]. At the same time, ALT is most closely related to liver fat accumulation [9], and is commonly used as a marker of NAFLD [10]. Previous studies have demonstrated that elevated ALT concentration is associated with increased risk of MetS [11,12,13,14,15,16,17,18,19]. Among above studies, the Hoorn Study and a Korean study both pointed out that ALT was correlated with metabolic syndrome independent of insulin resistance [14,27]. In addition, two Japanese studies and a Korean study shown that risk of metabolic syndrome increased with elevation in serum ALT level, even within reference range [15,16,17]. The present study demonstrated that serum ALT level within normal range was associated with MetS in a dose-response manner. Our results were in accordance with those of previous studies. The odds ratio values of MetS in the ALT quartiles 2–4 groups were 1.920 (95%CI: 1.619–2.277), 2.853 (95%CI: 2.407–3.381), and 4.171 (95%CI: 3.510–4.956) in males; 1.889 (95%CI: 1.671–2.136), 3.490 (95%CI: 3.095–3.935), and 5.593 (95%CI: 4.957–6.311) in females, respectively, compared with the ALT quartile 1 group.

A Korean cross-sectional survey showed that ALT level was associated with all components of MetS [14]. In addition, a prospective study conducted by Goessling *et al*. [27] has shown that ALT level, even within the normal range, was an independent predictor of incident diabetes. The present study demonstrated that ALT level within the reference range was correlated with waist circumference, TG level, and fasting blood glucose in both men and women, as well DBP in women after adjustment for BMI. Among them, the association between ALT level and TG was strongest.

Serum ALT concentration is a sensitive, cheap, simple and specific indicator for liver injury. The current upper normal limit of serum ALT concentration is 40 U/L. However, in the majority of uncomplicated obese and NAFLD cases, ALT is still in the normal range [20,21]. Goessling *et al*. [28] reported that ALT within the normal range was an independent predictor of MetS. Our study also had demonstrated that higher serum ALT level within the reference range was associated with increased prevalence of MetS. Therefore, current reference range of serum ALT level maybe underestimated the prevalence of subclinical liver disease, most commonly NAFLD and MetS. Prati *et al*. [29] recommended that upper normal limit of ALT was 30 U/L for men and 19 U/L for women. In our study, the optimal cutoff value of serum ALT concentration was 23.3 U/L for males and 16.0 U/L for females. The sensitivity for diagnosis of MetS respectively was 68.2% in males and 74.1% in females. The updated upper limit of ALT could help improve the sensitivity of detecting MetS and early identify the individuals at risk of developing MetS. But more prospective studies are needed to identify the Chinese optimal cutoff value of ALT.

The present study shown that ALT level decreased with age in males; however, in females, subjects in the lowest ALT quartile were younger than those in the other three groups. Polotsky reported that incidence of MetS increased substantially during perimenopause and early menopause. Postmenopausal women were at a higher risk of hypertension, atherogenic dyslipidemia, and diabetes as compared with their premenopausal counterparts [30]. Therefore, this could explain why the association of ALT with age was different in women compared to men.

The strengths of our study were the population-based design, large sample size, and excessive information on confounders. Our study had some limitations. Our study was a cross-sectional survey, and therefore we could not establish the causality. We didn’t measure the HOMA index, and thus we couldn’t determine the association between serum ALT level and MetS after adjustment for insulin resistance.

## 5. Conclusions

In conclusion, our study demonstrated that elevated serum ALT level, even within the reference range, was associated with increased odds of MetS in middle-aged and elderly Chinese men and women. Further, prospective studies are needed to clarify the importance of serum ALT level in development of MetS.
